# Supplementary material for: Power and interest levels in safely managed sanitation services in Zambia: A stakeholder mapping
Source: PLoS One. 2025 Nov 4;20(11):e0335130. doi: 10.1371/journal.pone.0335130 (PMC12585081; doi:10.1371/journal.pone.0335130)
Supplement: Attachment — Submitted filename: Response to Reviewers.docx [file pone.0335130.s003.docx]

**Responses to Editor and Reviewer’s comments**

**A**: **Academic Editor’s comments**

1. Please review your reference list to ensure that it is complete and correct. If you have cited papers that have been retracted, please include the rationale for doing so in the manuscript text, or remove these references and replace them with relevant current references. Any changes to the reference list should be mentioned in the rebuttal letter that accompanies your revised manuscript. If you need to cite a retracted article, indicate the article’s retracted status in the References list and also include a citation and full reference for the retraction notice.

**B**: **Reviewer’s Responses to Questions**

Journal Requirements:

If the reviewer comments include a recommendation to cite specific previously published works, please review and evaluate these publications to determine whether they are relevant and should be cited. There is no requirement to cite these works unless the editor has indicated otherwise. 

Please review your reference list to ensure that it is complete and correct. If you have cited papers that have been retracted, please include the rationale for doing so in the manuscript text, or remove these references and replace them with relevant current references. Any changes to the reference list should be mentioned in the rebuttal letter that accompanies your revised manuscript. If you need to cite a retracted article, indicate the article’s retracted status in the References list and also include a citation and full reference for the retraction notice.

***Response****: Thank you for the suggestion. We have since reviewed the reference list to ensure that it is complete and correct. As part of the revisions, we have added/updated the DOI and URL where applicable/available to improve accessibility of the cited sources. We have also reviewed and evaluated the cited articles and confirmed that they are still relevant to the study.*

[Note: HTML markup is below. Please do not edit.]

Reviewers' comments:

Reviewer's Responses to Questions

**Comments to the Author**

1. If the authors have adequately addressed your comments raised in a previous round of review and you feel that this manuscript is now acceptable for publication, you may indicate that here to bypass the “Comments to the Author” section, enter your conflict of interest statement in the “Confidential to Editor” section, and submit your "Accept" recommendation.

Reviewer #2: All comments have been addressed

***Response****: Thank you for the positive feedback.*

2. Is the manuscript technically sound, and do the data support the conclusions?

The manuscript must describe a technically sound piece of scientific research with data that supports the conclusions. Experiments must have been conducted rigorously, with appropriate controls, replication, and sample sizes. The conclusions must be drawn appropriately based on the data presented.

Reviewer #2: Yes

***Response****: Thank you for the positive feedback.*

3. Has the statistical analysis been performed appropriately and rigorously?

Reviewer #2: N/A

***Response****: Thank you for the positive feedback.*

4. Have the authors made all data underlying the findings in their manuscript fully available?

The [PLOS Data policy](http://track.editorialmanager.com/CL0/http:%2F%2Fwww.plosone.org%2Fstatic%2Fpolicies.action%23sharing/1/010f019880abc2cb-42f09e63-4f36-4f71-bff0-ddfe5c268691-000000/DA3BYD8dnI3-aY4i0Mp7sFr0LDle_e5iqD4eA3tpfG0=223) requires authors to make all data underlying the findings described in their manuscript fully available without restriction, with rare exception (please refer to the Data Availability Statement in the manuscript PDF file). The data should be provided as part of the manuscript or its supporting information, or deposited to a public repository. For example, in addition to summary statistics, the data points behind means, medians and variance measures should be available. If there are restrictions on publicly sharing data—e.g. participant privacy or use of data from a third party—those must be specified.

Reviewer #2: Yes

***Response****: Thank you for the positive feedback.*

5. Is the manuscript presented in an intelligible fashion and written in standard English?

PLOS ONE does not copyedit accepted manuscripts, so the language in submitted articles must be clear, correct, and unambiguous. Any typographical or grammatical errors should be corrected at revision, so please note any specific errors here.

Reviewer #2: Yes

***Response****: Thank you for the positive feedback.*

6. Review Comments to the Author

Please use the space provided to explain your answers to the questions above. You may also include additional comments for the author, including concerns about dual publication, research ethics, or publication ethics. (Please upload your review as an attachment if it exceeds 20,000 characters)

Reviewer #2: The revised manuscript demonstrates a strong effort to address and respond to my previous comments. Key structural, conceptual, and linguistic revisions were incorporated in several sections. However, some minor adjustments would improve the manuscript. For example: in results section, including a diagram of stakeholder categories by quadrant would greatly enhance the reader’s comprehension.

***Response****: Thank you for the feedback. We have inserted a table highlighting the grouped sanitation stakeholder per quadrant as advised in lines 227 to 231 as indicated below.*

*We further grouped the stakeholders per quadrant as demonstrated in table 4 below:*

*Table 4: Stakeholder categories per quadrant*
